# Gametocytaemia after Drug Treatment of Asymptomatic Plasmodium falciparum

**DOI:** 10.1371/journal.pctr.0010020

**Published:** 2006-08-18

**Authors:** Samuel Dunyo, Paul Milligan, Tansy Edwards, Colin Sutherland, Geoffrey Targett, Margaret Pinder

**Affiliations:** 1 Medical Research Council Laboratories, Banjul, Gambia; 2 London School of Hygiene and Tropical Medicine, London, United Kingdom

## Abstract

**Objectives:**

Treatment of Plasmodium falciparum malaria with sulfadoxine-pyrimethamine (SP) is followed by a sharp rise in the prevalence and density of gametocytes. We did a randomized trial to determine the effect of treatment of asymptomatic infections with SP or SP plus one dose of artesunate (SP+AS) on gametocyte carriage.

**Design:**

The study was a three-arm open-label randomized trial. We randomized asymptomatic carriers of P. falciparum to receive antimalarial treatment or placebo, and recorded the prevalence and density of gametocytes over the next 2 mo.

**Setting:**

The trial was conducted during the dry (low malaria transmission) season in four rural villages in Gambia.

**Participants:**

Participants were adults and children aged over 6 mo with asexual P. falciparum infection and confirmed free of clinical symptoms of malaria over a 2-d screening period.

**Interventions:**

Participants were randomized to receive a single dose of SP or SP+AS or placebo.

**Outcome Measures:**

The outcome measures were the presence of gametocytes 7 and 56 d after treatment, and the duration and density of gametocytaemia over 2 mo.

**Results:**

In total, 372 asymptomatic carriers were randomized. Gametocyte prevalence on day 7 was 10.5% in the placebo group, 11.2% in the SP group (risk difference to placebo 0.7%, 95% confidence interval −7.4% to 8.7%, *p* = 0.87), and 7.1% in the SP+AS group (risk difference to placebo 4.1%, 95% confidence interval −3.3% to 12%, *p =* 0.28). By day 56, gametocyte prevalence was 13% in the placebo group and 2% in both drug-treated groups. Gametocyte carriage (the area under the curve of gametocyte density versus time), was reduced by 71% in the SP group, and by 74% in the SP+AS group, compared to placebo. Gametocyte carriage varied with age and was greater among children under 15 than among adults.

**Conclusions:**

Treatment of asymptomatic carriers of P. falciparum with SP does not increase gametocyte carriage or density. Effective treatment of asexual parasitaemia in the dry season reduces gametocyte carriage to very low levels after 4 wk.

## Editorial Commentary

**Background:** During the life cycle of the Plasmodium falciparum malaria parasite, some parasites differentiate into sexual stages (gametocytes) in the blood of infected people. Gametocytes are the transmissible stage of the parasite that can infect the mosquito when it takes a blood meal from an infected person. In people with clinical signs of malaria, treatment with commonly used antimalarial drugs such as chloroquine or sulfadoxine-pyrimethamine is often followed by a rise in the numbers of gametocytes in the blood, but it is not known to what extent this is caused by the drugs themselves. The effects of different antimalarial treatments on gametocytes have generally not been well studied in trials, and particularly not in individuals who are infected with malaria but don't show clinical signs (i.e., asymptomatic carriers). However, it is important to know the effects of different malaria drugs on gametocytes, because this will help predict how parasites are likely to evolve resistance to drugs. In particular, the effects on asymptomatic carriers are interesting because of the potential benefit of giving preventive treatments to all children or adults (whether or not they have symptoms) for malaria control. The trial from Gambia reported here was conducted in adults and children over 6 mo of age who were infected with P. falciparum malaria but free from clinical symptoms. Participants were randomized to receive either sulfadoxine-pyrimethamine, sulfadoxine-pyrimethamine and artesunate, or placebo. The researchers wanted to look at the effect of these treatments on numbers of gametocytes in the participants' blood samples seven days after treatment (the primary outcome) and then up to 56 days after treatment.

**What this trial shows:** Seven days after treatment, the researchers found that the proportion of participants carrying malaria gametocytes in the groups treated with sulfadoxine-pyrimethamine or sulfadoxine-pyrimethamine and artesunate was similar to the proportion in the placebo group. A secondary objective of the study was to look at overall gametocyte load over 56 days following treatment, by measuring the area under the curve plotting gametocyte density against time. For this secondary objective, the researchers found that gametocyte load was highest in the placebo group, and substantially lower in the groups treated with sulfadoxine-pyrimethamine or sulfadoxine-pyrimethamine and artesunate. Therefore, there was no evidence that treatment with sulfadoxine-pyrimethamine increased gametocytes in the blood of children infected with malaria but without symptoms.

**Strengths and limitations:** In this trial, screening tests to detect malaria infection and symptoms were done twice prior to enrolling participants into the trial, with a two-day window between tests. This procedure increased the chance of detecting individuals with clinical malaria so they could be excluded from the trial and treated for malaria. The use of a placebo arm in the trial, which adds to the strength of the study, could therefore be defended ethically. The conduct of this trial in a region with seasonal transmission of malaria limits the extent to which the findings can be generalized to other regions.

**Contribution to the evidence:** This trial provides data on the extent to which treatment with sulfadoxine-pyrimethamine or sulfadoxine-pyrimethamine and artesunate affects gametocyte levels in people infected with malaria, but without symptoms. It had previously been thought that sulfadoxine-pyrimethamine increased gametocyte carriage, but this had not been formally tested in a placebo-controlled study. The results show that both treatments reduce overall gametocyte load to very low levels over a period of 56 days after treatment, as compared to placebo.

## INTRODUCTION

In sub-Saharan Africa, malaria caused by Plasmodium falciparum is responsible for 300 million clinical cases and up to 1 million deaths annually, mainly among children under 5 y of age. Treatment of P. falciparum malaria with commonly used antimalarial drugs such as chloroquine or sulfadoxine-pyrimethamine (SP) is followed by a marked increase in the density of gametocytes, the parasite stage that infects mosquitoes [1–5]. This density increase may reflect drug-induced release of sequestered gametocytes into the peripheral circulation after treatment with SP but could also be explained by the natural wave of gametocytaemia in an acute infection, against which antimalarial drugs have varying impact [5,6]; this issue has not been investigated experimentally. Drug-induced release of gametocytes could enhance transmission of resistant parasites and would argue against the use of SP, especially for intermittent preventive treatment [7,8]. The effects of antimalarial drugs on gametocytes and their infectiousness to vector mosquitoes have been mainly studied either in vitro or in clinical cases [1–5,9–14], and so the effect of antimalarial treatment on gametocyte carriage has not been evaluated in a randomized placebo-controlled study. Treatment with artemisinin derivatives in combination with other drugs results in lower gametocyte carriage rates and density, and reduced infectiousness of treated individuals compared to non-artemisinin regimens [3,4,9,11,14,15]. These properties suggest artemisinin-based combination therapies could be effective tools for intermittent treatment strategies (e.g., [16]), but few data exist on the dynamics of gametocytaemia, both with and without drug treatment, in individuals who are infected but without symptoms [17]. To investigate this, we randomized individuals who had P. falciparum infection without clinical symptoms of malaria to treatment with SP, a combination of SP plus one dose of artesunate (SP+AS), or placebo, to determine the effects of treatment on gametocyte prevalence, duration of gametocyte carriage, and gametocyte density.

## METHODS

### Participants

The climate in Gambia is characterised by a long dry season from mid-October to mid-June followed by a short rainy season. Malaria is predominantly caused by *P. falciparum,* and occasionally by P. malariae and *P. ovale,* and transmission is highly seasonal, with most morbidity due to malaria occurring from September to November [18]. The study was conducted in four villages located west of Farafenni in the North Bank Division of Gambia. The total population of the villages, which are included in the Farafenni Demographic Surveillance System, was 2,300. Meetings were held in the villages to explain the objectives of the study and to seek the consent of the community leaders and villagers. All residents aged 6 mo and above were identified using the Demographic Surveillance System database and screened in order of Demographic Surveillance System number for participation if they, or their accompanying parents or guardians in the case of children under 16 y, gave written consent. Screening continued until the required quota of eligible participants was obtained for randomizaton. Gambia Government/Medical Research Council Joint Ethics Committee approved the study. Screening took place from May to the end of June in 2001. Eligible participants were asked about symptoms suggestive of malaria and were clinically examined. Axillary temperature was measured using a digital thermometer and, in children 2–9 y, spleen size was determined. Finger prick blood samples were taken for packed cell volume (PCV) determination and malaria microscopy. Two days later (day 0 [D0]), the results were given to the participants and the interview and clinical examination repeated for those who had asexual parasitaemia and had been free of symptoms. Individuals with at least two asexual parasites per 50 high-powered fields (HPFs) (20 asexual parasites per microlitre of blood) without pyrexia (temperature ≥ 37.5 °C) or other symptoms suggestive of malaria at the first and second screening were enrolled into the study as asymptomatic malaria cases. The parasitaemia cut-off (20 parasites per microlitre of blood) was chosen to minimise the risk of enrolling false positives [19]. Exclusion criteria were pregnancy, weight less than 5 kg, symptoms or signs of other diseases at the first and/or second screening, and history of hypersensitivity to any of the study drugs or their use within the past 4 wk. Anaemia was not an exclusion criterion, but participants with PCV less than 33% were given a course of iron tablets. Chloroquine was the only antimalarial drug available in the villages and is dispensed by village health workers, but there was little chloroquine prescription in the villages at the time of the year that enrolment took place.

### Interventions

Participants enrolled were randomized to receive SP alone, SP+AS, or placebo. Participants who had symptomatic malaria at screening were not randomized but were treated with SP+AS and followed up similarly, in order to check that the normal pattern of gametocyte carriage after treatment in clinical cases was observed. Open-label SP (Pharmamed, Zejtun, Malta), artesunate (manufactured by Guilin Pharmaceutical Works and supplied by Sanofi, Paris, France), and calcium (Aegis, Lefkosia, Cyprus) were used.

### Objectives

We wanted to determine the effect of treatment with SP alone and with a single dose of artesunate on gametocyte carriage, in people with P. falciparum infection during the low-transmission season.

### Outcomes

The primary outcomes were gametocyte prevalence and density on D7, and secondary outcomes were gametocyte prevalence on D56 after treatment, and gametocyte carriage, defined as the area under the curve (AUC) of gametocyte density against time. Participants were visited at home by field workers on D3, D7, D14, D28, and D56 post-treatment to check that the person was well, to record self-reported adverse events, and to collect finger prick blood for microscopy. The study was not blinded, but field workers and laboratory assistants responsible for microscopy did not have access to treatment allocation data and therefore were likely to be unaware of the treatment received by participants. Two thick blood films were prepared from each individual, dried overnight, and stained with Giemsa, and 200 HPFs were examined independently by two microscopists for asexual and sexual stage parasites. Where there was a discrepancy the slide was read a third time, by another microscopist. During screening, one blood film was stained with Field's stain, and 50 HPFs were examined to identify infected individuals for enrolment. PCV was determined by collecting finger prick blood samples into heparinised capillary tubes and spinning them using a micro-haematocrit centrifuge (Hawksley, Lancing, England).

### Sample Size

The primary endpoint was gametocyte prevalence on D7. Assuming a gametocyte rate in the placebo group of 20%, a sample size of 120 individuals per group would allow over 80% power to detect an increase to 40% in the SP-alone group and a decrease to 5% or less in the SP+AS group, allowing for 10% drop-out and using a significance level of 0.025 (to allow for two primary comparisons with an overall type 1 error rate of 0.05).

### Randomization

The randomization sequence was generated in Stata (Statacorp, College Station, Texas, United States) using restricted randomization with a block size of 12. Treatment allocation was determined by opening pre-prepared numbered opaque randomization envelopes in sequence by a study nurse who took no part in participant selection or evaluation, but who acted as the drug dispenser throughout the trial. SP was administered as 25 mg/kg sulfadoxine/1.25 mg/kg pyrimethamine for children under 50 kg, while adults and children weighing 50 kg or more were given three tablets (each tablet contained 500 mg of sulfadoxine and 25 mg of pyrimethamine) . In the SP+AS group, artesunate was given at the same time as SP at a dose of 200 mg (four tablets at 50 mg) for adults and for children weighing 50 kg or more, while children under 50 kg received 4 mg/kg body weight. Calcium tablets were given as placebo. Children were observed for 1 h after treatment to ensure that the drug was not vomited, and treatment was repeated in those who vomited the first dose. Treatment failures in the SP and SP+AS groups were to receive rescue medication with quinine while those in the placebo group were to be rescued with chloroquine and SP (which is highly effective in Gambia [3]).

### Statistical Methods

Data were double entered and validated. Analysis was done using Stata. Fractional polynomial logistic regression [20] was used to obtain a smoothed estimate of the proportion of gametocyte carriers as a function of age. Parasite density was calculated by assuming one parasite per HPF to be equivalent to 500 parasites per microlitre blood [21]. Primary analysis was by intention to treat. Gametocyte prevalence was compared using the chi-squared test and gametocyte densities using one-way analysis of variance on log-transformed densities. *p*-Values are two-sided. We summarised the information on gametocyte carriage by calculating the AUC of the gametocyte density against time, a measure of transmission potential. This is a weighted sum of the gametocyte densities, with weights proportional to the difference in time between adjacent sampling points. Individuals with missing data were included in the analysis, with the AUC weighted according to the number of non-missing sampling points. Similarly, an AUC was calculated for the binary variable (gametocyte positive, 1, or negative, 0) against time. The mean of this AUC is the area under the curve of the proportion positive plotted against time, and is a measure of the average duration of gametocyte carriage. We compared the AUC between treatment groups using the Kruskal-Wallis test and between age groups using a non-parametric test for trend [22]; both tests were adjusted for tied values.

## RESULTS

### Recruitment and Participant Flow

**Figure 1:**
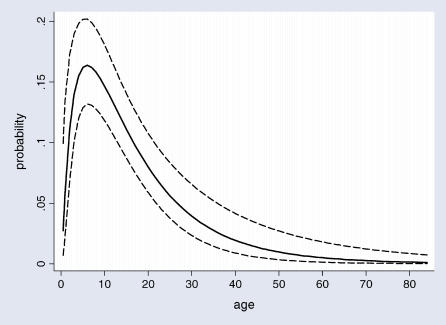
Smoothed Estimate of Gametocyte Prevalence in Relation to Age (in Years) among 1,048 Children and Adults Who Were Screened Dashed lines indicate 95% CIs.

A total of 1,184 participants were screened in May and June 2001. Of these, 136 were excluded (78 had missing parasitological data, finger prick samples were not collected from 19 women who were pregnant, and 39 participants had clinical P. falciparum malaria). Of the remaining 1,048 participants, representing 46% (1,048/2,300) of the population of the study villages, 99 (9.4%) had gametocytaemia with a geometric mean density of 6.1 (95% confidence interval [CI] 5.0 to 7.5) gametocytes per microlitre. A total of 616/1,048 (59%) did not have detectable asexual parasitaemia, and most of these (97.4%) were gametocyte negative. Gametocyte prevalence was high among children under 15 y and then decreased with increasing age, and gametocyte prevalence was similar in males and females and in each of the four villages (Table 1). Gametocyte prevalence in relation to age is shown in Figure 1.

**Table 1:**
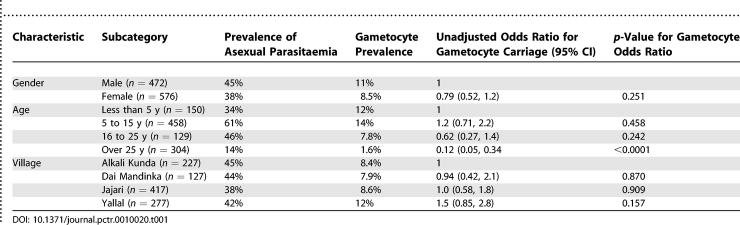
Prevalence of Asexual Parasitaemia and Gametocytes in Children and Adults Who Were Screened

**Figure 2:**
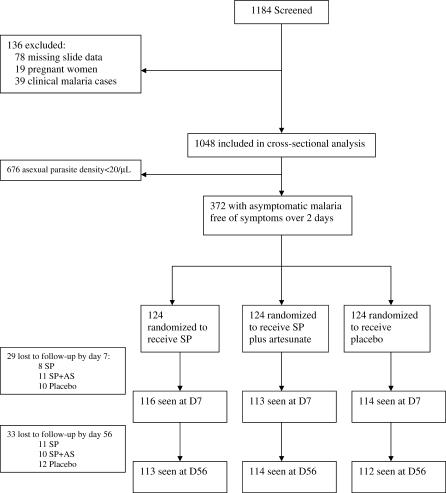
Trial Profile

Out of 1,148 screened individuals, 372 had asymptomatic P. falciparum infection above 20 parasites per microlitre and were enrolled into the trial (Figure 2). In addition, 39 clinical cases that were not randomized were treated with SP+AS and followed up. The randomized groups were similar in demographic and clinical characteristics and asexual parasite density and gametocyte rates at baseline (Table 2). On completion of follow-up (D56), parasitological data were available for 339 (91%) individuals in the trial. During the 56 d of follow-up there were no cases of clinical malaria and no adverse events were reported in any trial participants.

**Table 2:**
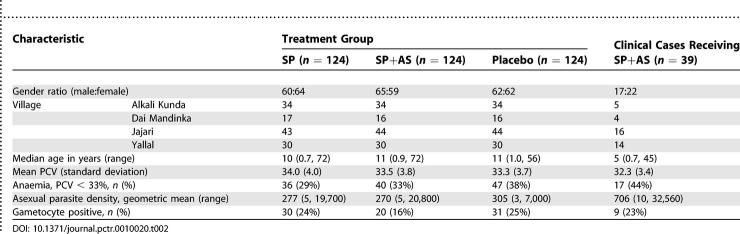
Baseline Characteristics of Trial Participants and Clinical Cases

### Outcomes and Estimation: The Effect of Treatment on Asexual Parasitaemia and Gametocytaemia

**Figure 3:**
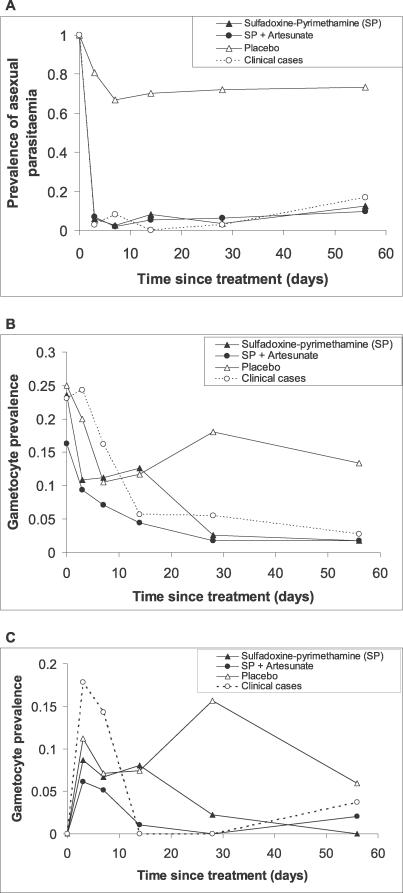
Prevalence of Asexual Parasitaemia and Gametocytes after Treatment (A) Prevalence of asexual parasitaemia over the 2 mo following treatment. The profile for the 39 clinical cases, who were treated with SP+AS, is also shown. (B) Prevalence of gametocytes. (C) Prevalence of gametocytes in the subset of participants who were gametocyte negative on D0.

Asexual parasite clearance was similar in the SP and SP+AS groups (Figure 3A). Gametocyte rates in the SP and placebo groups were similar on D3, D7, and D14. On D7, the prevalence of gametocyte carriage was 10.5% in the placebo group and 11.2% in the SP group (risk difference 0.7%, 95% CI −7.4% to 8.7%, *p* = 0.87), and 7.1% in the SP+AS group (difference between SP+AS and SP groups, 4.1%, 95%CI −3.3% to 12%, *p =* 0.28). Gametocyte prevalence showed a steady decline in the active drug treatment groups, and by D28, gametocyte prevalence was less than 3% in the SP and SP+AS groups (Figure 3B). In contrast, gametocyte prevalence in the clinical cases had increased by D3. The AUC index of gametocyte carriage (Table 3) was 62 in the placebo group; treatment with SP reduced this index to 18 (a 71% reduction, *p <* 0.001, Kruskal-Wallis test), and addition of AS reduced this index to 16 (a 74% reduction from the value in the placebo group, *p <* 0.001, and only slightly lower than the SP group, *p =* 0.02). In the clinical cases, the AUC gametocyte index was 147, significantly greater than the asymptomatic group treated with the same drug regimen (*p =* 0.02) but not significantly different from the placebo group (*p =* 0.32).

**Table 3:**
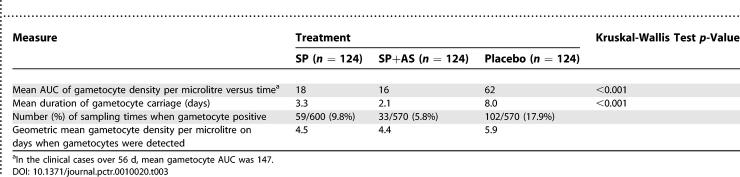
Effect of Treatment on Gametocyte Carriage

Among the 290 randomized participants who were gametocyte negative at enrolment, gametocyte prevalences on D3, D7, and D14 were similar in the SP and placebo groups (Figure 3C), suggesting SP has no effect on the release of tissue-sequestered gametocytes into the peripheral circulation.

Mean AUC gametocyte index varied with age and was greatest among children under 15 y; this pattern was associated with greater asexual parasite densities in this age group at enrolment (Table 4). The average gametocyte AUC index for the population of the study villages—obtained as a weighted mean of the age-specific gametocyte AUC values for the placebo group, taking account of the age distribution and asexual parasite prevalences in Table 1 and assuming negligible gametocyte carriage among asexual negative individuals—was 25; if gametocyte carriage among adults is ignored, this mean value becomes 23, indicating that gametocyte carriage among adults contributes only a small percentage to the overall value.

**Table 4:**
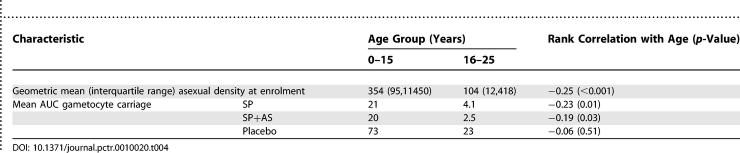
Gametocyte Carriage in Relation to Age and Asexual Parasitaemia at Enrolment

## DISCUSSION

### Interpretation

In areas of seasonal malaria transmission, intermittent treatment of children with antimalarial drugs or some form of chemoprophylaxis may have a role in malaria prevention [8,16,18,23]. Mass treatment (single or repeated administrations of antimalarial drugs to all members of the population, usually during the dry season, in an attempt to reduce transmission) has not proved a useful strategy [24] but might be effective in areas where there is a very short transmission season [25]. In recent randomized trials of these approaches, SP has been used alone or in combination with artesunate. We investigated the effects of antimalarial treatment with these drugs on circulating gametocytes in volunteers with asymptomatic P. falciparum infection, and we found no evidence that SP treatment enhances emergence of gametocytes into the peripheral blood, but rather that, during the dry season, effective treatment with SP or SP+AS virtually eliminates gametocyte carriage over time. Gametocyte carriage was not significantly reduced further by the addition of a single dose of artesunate; three doses may be more effective [3]. Administration of antimalarial drugs through intermittent or mass treatment strategies carries the risk that selection for drug-resistant genotypes will be increased. This risk needs careful evaluation, but our study shows that preventive treatment of asymptomatic individuals with SP alone or in combination with artesunate in the dry season leads to a substantial reduction in gametocyte carriage and therefore may reduce transmission from treated individuals. In our study, the AUC index of gametocyte carriage was substantially higher in children under 15 y of age than in adults. This finding is compatible with acquisition of anti-gametocyte immunity [17] but could be explained by greater ability to control asexual parasitaemia in adults [26]. Children under 15 y carry gametocytes for longer and at higher densities than adults, but other factors such as differences in the degree of exposure to biting mosquitoes and the greater skin area of adults make it difficult to estimate the relative contribution of adults and children to transmission.

### Generalisability

Our study, conducted in the dry season in asymptomatic carriers, does not rule out the possibility that in acute clinical cases, with much greater parasite densities, treatment with SP causes some release of sequestered gametocytes, but our data are compatible with the explanation suggested by Butcher [6], that the rise in gametocytaemia observed immediately after SP treatment in clinical cases [3–5,27] is most likely explained by the natural wave of gametocytaemia in an acute infection, against which SP has no impact, but which is time-limited due to the eradication of the asexual parasite biomass.

### Overall Evidence

Treatment reduces gametocyte carriage to very low levels over a period of 1 mo; mass drug administration of SP+AS in the study area in June 1999 did not reduce overall malaria incidence despite achieving relatively high coverage [24], and while low levels of gametocyte carriage may be sufficient to sustain transmission, earlier treatment might have had a greater impact.

## SUPPORTING INFORMATION

CONSORT Checklist Click here for additional data file. (48 KB DOC)

Trial Protocol Click here for additional data file. (229 KB DOC)

## Abbreviations

AUC: area under the curve
CI: confidence interval
D[number]: day [number]
HPF: high-powered field
PCV: packed cell volume
SP: sulfadoxine-pyrimethamine
SP+AS: sulfadoxine-pyrimethamine plus one dose of artesunate
