# Supplementary material for: Gametocytaemia after Drug Treatment of Asymptomatic Plasmodium falciparum
Source: PLoS Clin Trials. 2006 Aug 18;1(4):e20. doi: 10.1371/journal.pctr.0010020 (PMC1851719; doi:10.1371/journal.pctr.0010020)
Supplement: Trial Protocol [file pctr.0010020.sd002.doc]

# Medical Research Council Laboratories, Fajara

**Application to undertake a research project**

**__________________________________________________________________________**

#### A Summary Information

**A1 Title of project**

Please choose a short clear title for ease of reference and identification in future.

**Gametocytaemia after drug treatment of asymptomatic *Plasmodium falciparum* infection and its implications for transmission control**

**A2 Investigators (Principal Investigator first)**

Please list all investigators and collaborators and attach CV if the principal investigator is unknown to the Committee

| **Name** | **Institution** | **Position** |
| --- | --- | --- |
| Samuel Dunyo | MRC, The Gambia | Clinical Epidemiologist, Malaria Programme |
| Margaret Pinder | MRC, The Gambia | Head, Malaria Programme |
| Gijs Walraven | MRC, The Gambia | Head, MRC Station Farafenni |
| Maimuna Sowe | MRC, The Gambia | Data Manager, Farafenni |
| Kalifa Bojang | MRC, The Gambia | Clinical Scientist, Malaria Programme |
| Paul Milligan | MRC, The Gambia | Statistician |
| Colin Sutherland | London Sch Hyg & Trop Med | Scientist |
| Geoffrey Targett | London Sch Hyg & Trop Med | Professor |
|  |  |  |

**Who will introduce the proposal at SCC?**

Margaret Pinder

**A3 Location(s) of research**

Please list all the places where the research will take place including field sites or health facilities

MRC Farafenni, and the villages: Yallal, Alkali Kunda, Dai Mandinka and Jajari

**A4 Proposed start date and duration in months**

In giving a date please bear in mind the time scale for decision-making by SCC, the Ethical Committee and any other institutions whose agreement is needed, and the time needed to organise the resources required.

March 2001 for three months

__________________________________________________________________________

**A5 Reference (office use)**

**Scientific Co-ordinating Committee No ....……**

**Ethical Committee No ....……**

**A6 Summary of project, long term objectives and specific aims (not more than 200 words)**

This section is very helpful to the Committees in determining quickly the main features of the study, and should be as clear and concise as possible. It should cover the key objectives and endpoints and, if the project is hypothesis driven, then the hypothesis should be stated here.

Knowledge of response of the human reservoir of malaria gametocytes (sexual forms) to chemotherapy and their subsequent infectivity to mosquitoes is crucial for reducing disease transmission through mass drug administration (MDA). Recent studies have shown that malaria treatment using the combination of Fansidar® (25 mg pyrimethamine plus 500 mg sulfadoxine) and artesunate result in lower gametocyte levels than following Fansidar® or chloroquine alone. Combinations including artemisinin derivatives therefore have potential for use in transmission reduction. Nevertheless, treatment with these drugs in symptomatic individuals is followed by an increase in gametocytaemia for a few weeks. Their effect on gametocytaemia in infected asymptomatic individuals is not known.

In an endemic area, only a small proportion of malaria infections result in acute illness. Many individuals have a low-level asymptomatic parasitaemia that may persist for months. In applying MDA, therefore, it is important to ensure that the treatment either reduces gametocytaemia in asymptomatic individuals, or if this increases an estimate of the duration is required. The aims of this study are therefore to 1) test the hypothesis that treatment of asymptomatic *Plasmodium falciparum* malaria infection with the combination of Fansidar® and artesunate reduces gametocyte prevalence whereas treatment with Fansidar® alone increases this 2) model the results for their impact in a MDA intervention in the study area.

Residents in four villages west of Farafenni will be screened and those with asymptomatic *P. falciparum* malaria infection will be randomised into three treatment groups to receive either Fansidar® alone, the combination of Fansidar® and artesunate or placebo. All the treated individuals will be visited at home on days 3, 7, 14, 28 and 56 post-treatment to collect finger prick blood thick films, and filter paper and Tri-reagent blood samples for malaria parasite detection by microscopy and molecular techniques, respectively. Our primary endpoint is the prevalence of gametocytaemia at day 7 after treatment. The secondary endpoints include the incidence and density of gametocytaemia during the follow-up period and risk factors for gametocytaemia.

**A7 Checklist/Signatures**

**Please complete the following checklist and comment as appropriate.** This section is designed to ensure that all the planning steps have been taken that are needed for successful project. For projects at the MRC Laboratories, Programme Heads will help visiting workers, and others preparing proposals at a distance, to ensure liaison with key individuals who need to be consulted locally.

1. **Has the project been discussed and cleared with the institutions in which research will be carried out including health services to which the study will need access?**

Yes

1. **Have all investigators and collaborators given their agreement to take part in the study as described?**

Yes

1. **Has a CV been attached for the principal investigator if unknown to the Committee?**

Not applicable

1. **Have ethical issues been addressed? Give details in section C.**

Yes

1. **Have safety issues been addressed? Please give details**

Standard safety procedures for the collection and handling of human blood samples will be adhered to.

1. **Will the project require data and/or materials to be taken out of The Gambia? If so please give details**

If the molecular techniques for parasite typing and gametocyte detection have not been established in The Gambia in time then the filter papers and samples treated with tri-reagent may be analysed in London.

1. **For projects to be carried out at MRC Laboratories: Have the following been consulted about the support services, resources and working space required?**

|  | **Consulted: Yes/No/Comment** |
| --- | --- |
| Laboratory Manager (including safety issues) |  |
| Director of Clinical Services |  |
| Head of Computing |  |
| Transport Manager |  |
| Finance Manager |  |
| Personnel Manager |  |
| Administrative Director |  |
| Other services – specify |  |

**Signature of principal investigator: Date:**

**__________________________________________________________________________**

**B Description of Project**

not more than 5 pages covering the following:

**B1 Background**

The background should show the relationship between the proposed study and the present state of knowledge and should reference previous work by the investigators and others. The results of any pilot experiments should be stated.

**Introduction**

Malaria is recognised as a major health problem in many developing countries, especially in Africa. Despite this realisation public health measures for its prevention and control are woefully inadequate. Strategies for control derive from our knowledge of the life cycle of transmission and disease. Malaria is transmitted when a feeding female anopheline mosquito inoculates sporozoites into the human host during a blood meal. After pre-erythrocytic schizogony in the hepatocytes, merozoites are liberated which immediately invade erythrocytes to begin the asexual cycle that can cause clinical malaria. After at least two asexual cycles a proportion of parasites develop into sexual forms (gametocytes) which are infective to mosquitoes and maintain the life cycle. Control measures directed at the vector such as use of residual insecticide sprays, reduction of breeding sources or use of insecticide impregnated bed nets have been difficult to maintain mainly because of logistical problems, the high cost and insecticide resistance.

Nowadays, the cornerstone of malaria control in Africa is early recognition and prompt treatment of clinical cases (WHO, 1993). Nevertheless, treatment of *Plasmodium falciparum* malaria with chloroquine or Fansidar® (25 mg pyrimethamine plus 500 mg sulfadoxine), the two most commonly used antimalarial drugs in Africa, results in high levels of gametocytes and this may enhance transmission (BUCKLING *et al*., 1999; ENOSSE *et al*., 2000; ROBERT *et al*., 2000; SEIDLEIN *et al*., 2000; TARGETT *et al*., 2001). The 8-aminoquinoline drugs such as primaquine are efficacious in destroying gametocytes but they also have oxidant properties so that patients are required to be screened for red cell defects (especially glucose-6-phosphate dehydrogenase (G6PD)-deficiency) that would render their cells susceptible to oxidant lysis (GREWAL, 1981). Nevertheless primaquine has been used in many G6PD-deficient patients with relatively little haemolysis (WHO, 1973). Artesmisinin derviatives are gametocytocidal and recent studies have shown that these drugs alone, or in combination with Fansidar® or benflumetol, are safe and result in lower gametocyte levels compared to Fansidar® or chloroquine alone (PRICE *et al*., 1996; SEIDLEIN *et al*., 1998; DOHERTY *et al*., 1999; SEIDLEIN *et al*., 2000). Following the large-scale introduction of artesunate for the treatment of malaria in endemic areas of Thailand, a gradual decline in the incidence of malaria was observed. This has been attributed to the reduction in gametocyte levels and, therefore, transmissibility (PRICE *et al*., 1996). Theoretically, therefore, mass administration of artemisinin derivatives in combination with a conventional antimalarial drug may lower transmission in areas of seasonal malaria such as the Gambia. A trial of Fansidar® combined with one dose of artesunate resulted in four-fold lower transmission of infection to mosquitoes compared to Fansidar® alone (TARGETT *et al*., 2001). Nevertheless, mass administration of this combination just before the malaria season in the Gambia showed no overall reduction in malaria incidence after two months (SEIDLEIN *et al*., manuscript in preparation).

The effect of antimalarial drugs on gametocytes has been mainly studied *in vitro* and in treated clinical (i.e. symptomatic) cases (PRICE *et al*., 1996, 1999; BUCKLING *et al*., 1999; SEIDLEIN *et al*., 1998, 2000; DOHERTY *et al*., 1999; ENOSSE *et al*., 2000; ROBERT *et al*., 2000; TARGETT *et al*, 2001). Several factors may trigger gametocytogenesis including the clinical manifestations of malaria so that even without drug treatment gametocyte levels rise in symptomatic cases with a peak of gametocytes occurring 7 to 10 days after the first appearance of asexual parasitaemia (THOMPSON, 1911, 1914; MILLER, 1958). Haemolysis of infected erythrocytes can also play a role (SHUTE & MARYON, 1951; SCHNEWEIS *et al*., 1991) and recent work indicates that enzymnes of host erythropoiesis are involved (PAUL *et al*., 2000). Thus the part played by antimalarial drugs in the increase in gametocyte rate and concentration after treatment is unclear. Hogh and her colleagues (1995) examined one aspect of this in Mozambique by treatment of gametocytaemic children, 30% of whom were symptomatic, with chloroquine or Fansidar®. They found that in these children gametocyte density decreased over a 28 day period after either drug, leading the authors to conclude that neither drug increased gametocytogenesis. The study did not address whether gametocyte production was triggered in infected non-gametocytic individuals, and there are several studies reporting high numbers of gametocytes present for several months after drug treatment in man and studies in murine models show increased gametocytogenesis after chloroquine treatment (BUTCHER, 1997).

There is thus a gap in our knowledge of the dynamics of gametocytaemia, both with and without drug treatment, in individuals who are infected but without symptoms. In an endemic area like rural Gambia, only a small proportion of the reservoir of malaria infections results in acute illness and thus represents only part of the source from which mosquitoes acquire infection. A recent study in Farafenni villages found that only 20.1% of infected individuals sought medical treatment (SEIDLEIN *et al*., submitted). The same study showed that prevalence of asymptomatic infection varied with age and season from 5 to 70%, and approximately 30% of the residents were parasitaemic at the end of the dry season. This latter parasite rate was confirmed in our dry-wet season study conducted in June 2000 (PINDER & JAWARA, ongoing study). Knowledge of the response of the human reservoir of *P. falciparum* to chemotherapy and their subsequent infectivity to mosquitoes is therefore crucial for targeting transmission reduction through mass drug administration (MDA) and for predicting the impact of MDA as a transmission-reducing measure.

In applying MDA in an area of seasonal malaria transmission such as The Gambia, it is important to know the duration of time for which treated individuals remain gametocytaemic and are potentially infectious for mosquitoes. MDA resulting in increased gametocytaemia at the start of the rainy season might favour transmission. The overall aims of the study are thus twofold, to examine the impact of drug treatment on gametocyte dynamics in individuals with asymptomatic *P. falciparum* infection and to assess the utility of these drug treatments for MDA.

**Study Objectives**

1. To compare the prevalence and density of gametocytaemia at day 7 after treatment of asymptomatic *P. falciparum* infection with Fansidar® or the combination of Fansidar® and single dose artesunate to that found in the placebo treated group.
2. To measure the incidence and density of gametocytaemia during the follow-up period and assess risk factors for gametocytaemia so as to develop mathematical models to predict the impact of MDA using these drugs as a transmission reducing measure.

### B2 Project description

This should cover project plan, time-scales, descriptions of methods, justification, analyses to be carried out, expected outcomes (see also B3 where particular details need to be set out and cross refer as necessary).

*Timetable of activities*

|  |  | | **Year 2001** | | | | | | | | | |
| --- | --- | --- | --- | --- | --- | --- | --- | --- | --- | --- | --- | --- |
|  | | **Activities** | | March | | Apr | | May | | Jun | | Jul |
|  | Proposal submission & response from SCC and Ethical Committees | | X | |  | |  | |  | |  | |
|  | Meeting in study villages | |  | | X | |  | |  | |  | |
|  | Screening of study participants | |  | | X | | X | |  | |  | |
|  | Treatment and follow-up surveys | |  | | X | | X | | X | |  | |
|  | Data management and report writing | |  | | X | | X | | X | | X | |

**Methods**

*Study area and study population*

The study will be conducted from March to May 2001 in four villages (Yallal, Alkali Kunda, Dai Mandinka and Jajari) located west of Farafenni. These are rural communities in which families live in clusters of houses called compounds. There is a continuous updating of the population in the villages as part of the demographic surveillance system of the MRC Field Station in Farafenni (HILL *et al*., 2000). It is therefore possible to generate a register of the usual residents for the study. The total population of the villages as at February 1, 2001 was 2300. Meetings will be held in the villages to explain the objectives and methods of the study in the local languages, the inconveniences and advantages involved and to answer questions from the residents. Consent of the villages will be obtained before the commencement of the study. Residents aged 6 months and above will be screened for participation in the study after they, or their accompanying parents/relatives, have signed a written informed consent form **(Appendix 1)**.

*Study design*

All eligible participants will be interviewed on day-2 for symptoms suggestive of malaria (parents or guardians will be interviewed in the case of children). A clinical examination including measurement of axillary temperature and spleen size will be conducted and finger prick blood samples taken for haematocrit and parasitological examination. Two drops of blood will also be collected into Tri-reagent tubes and onto filter paper for detection of gametocytes using molecular techniques (RT-PCR) and parasite typing, respectively (see below). Two thick blood films will be prepared, dried overnight, stained with Giemsa and examined for malaria parasites. Two days later (day0) results of the microscopy will be given to the participants. A second interview for symptoms and clinical examination (including measurement of axillary temperature) will be conducted for those with malaria parasites. Individuals with *P. falciparum* infection of at least 2/50HPF and without symptoms suggestive of malaria or pyrexia (axillary temperature ≥ 37.5°C) at the first and second screening will be recruited into the study. They will be randomised into three groups to receive either Fansidar® alone, the combination of Fansidar® and single dose artesunate or placebo. All individuals with clinical and parasitological evidence of malaria will be treated with Fansidar® and single dose artesunate as we have previously found this to be a good treatment for clinical malaria (Von SEIDLEIN *et al.*, 1998). These symptomatic individuals will be followed in a similar manner to the rest of the cohort and analysed separately. Pregnant women, children <5 kg, individuals with symptoms and signs of other disease at the first and/or second screening, evidence of antimalarial use, history of hypersensitivity to any of the study drugs or their use within the past 4 weeks will be excluded from the study. Chloroquine is the only antimalarial available in the villages and is dispensed by village health workers (VHW's). Urine samples will therefore be collected on day-2 for detection of previous chloroquine intake using the modified Saker-Solomons test (MOUNT *et al.*, 1989).

*Randomisation and drug administration*

Selected study participants will be assigned by block randomisation to three groups. Group 1 will receive Fansidar® alone, group 2 will be treated with Fansidar® plus single dose artesunate and group 3 will receive placebo (lactose) tablets. Adults and those weighing ≥ 50 kg will be treated with 3 tablets of Fansidar® while children will receive half a tablet if their body weight is ≤10 kg and an additional quarter tablet for every 5-kg increase in weight. In the combined treatment group adults and those weighing ≥ 50 kg will receive additional 4 tablets of artesunate (50 mg tablet size) while children will be given 4 mg/kg of artesunate. All treatment will be administered by the PI. Children will be observed for 30 minutes after treatment to ensure that the drug is not vomited. Should this occur treatment would be repeated. Field workers will visit all participants at home on days 3, 7, 14, 28 and 56 post-treatment to record any symptoms, self-reported adverse reactions and to collect finger prick blood for microscopy and molecular assays for gametocytes and parasite typing. The field workers and laboratory assistants responsible for microscopy will be blinded to the treatment received by participants. Participants in the Fansidar® and Fansidar® plus single dose artesunate groups who present with symptoms and parasitological confirmation of malaria during the follow-up period will receive rescue medication with quinine while those in the control group will be rescued with chloroquine and Fansidar®. At the end of the study individuals with parasitaemia in the control group will be treated with chloroquine and Fansidar®.

# *Detection of sub-patent parasitaemia using RT-PCR*

Two to three drops of blood will be collected into tubes containing 200μl of Tri-reagent. This is a commercial based combination of guanidine-iso-thiocyanate and phenol that stabilises mRNA. The mRNA will be extracted using standard procedures. After extraction, the product will be treated with a mixture of DNA-ase and restriction enzymes specific to the fragments to be amplified to ensure the preparation is DNA free. A primary RT-PCR will be performed using primers specific for RESA, an asexual stage specific protein, and Pfs 16, a sexual stage specific protein. This is followed by a second, nested PCR that allows detection of very low levels of the product (Sutherland et al unpublished). Products are visualised by gel electrophoresis.

*Detection of treatment failures*

Drops of blood collected onto filter paper will be stored dry at +4° for subsequent parasite PCR typing using MSP1, MSP2 and GLURP polymorphisms to detect treatment failures.

*Data analysis*

The data will be double entered, validated and analysed using Epinfo and STATA computer software programmes, respectively. Variables to be considered in the analysis will be:

1. age and sex of study participants,
2. the prevalence and density of asexual parasitaemia and gametocytaemia before treatment,
3. the prevalence, incidence and density of gametocytaemia at the various time points after treatment,
4. prevalence and density of asexual parasitaemia at the various time points after treatment, and
5. influence of symptoms on gametocytaemia in individuals treated with Fansidar® artesunate

Discrete data will be compared using χ² while differences in parasite densities will be compared using the one-way ANOVA. Variables listed above will be fitted into a mathematical model to predict the impact of the various treatments on transmission.

**References**

Buckling, A., Ranford-Cartwright, C.L., Miles, A. & Read, A.F. (1999). Chloroquine increases *Plasmodium falciparum* gametocytogenesis *in vivo. Parasitology,* 118, 339-346.

Butcher, G.A. (1997). Antimalarial drugs and the mosquito transmission of *Plasmodium*. *International Journal of Parasitology,* 27, 975-987.

Doherty, J.F., Sadiq, A.D., Bayo, L., Alloueche, A., Olliaro, P., Milligan, P., von Seidlein, L. & Pinder, M. (1999). A randomised safety and tolerability trial of artesunate plus sulfadoxine-pyrimethamine versus sulfadoxine-pyrimethamine alone for the treatment of uncomplicated malaria in Gambian children. *Transactions of the Royal Society of Tropical Medicine and Hygiene*, 93, 543-546.

Enosse, S., Butcher, G.A., Margos, G., Mendoza, J., Sinden, R.E. & Høgh. B. (2000). The mosquito transmission of malaria: the effects of atovaquone-proguanil (Malarone™) and chloroquine. *Transactions of the Royal Society of Tropical Medicine and Hygiene*, 94, 77-82.

Greenwood, B.M. & Armstrong, R.M. (1991). Comparison of two simple methods for determining malaria parasite density. *Transactions of the Royal Society of Tropical Medicine and Hygiene*, 85, 186-188.

Grewal, R.S. (1981). Pharmacology of 8-aminoquinolines. *Bulletin of the World Health Organization*, 59, 397-406.

Hill, A.G., Macleod, W.B., Joof, D., Gomez, P., Ratcliffe, A.A. & Walraven, W. (2000). Decline of mortality in children in rural Gambia: the influence of village-level Primary Health Care. *Tropical Medicine and International Health,* 5, 107-118.

Hogh B, Thompson R, Hetzel C, Fleck SL, Kruse NA, Jones I, Dgedge M, Barreto J, Sinden RE (1995). Specific and nonspecific responses to *Plasmodium falciparum* blood-stage parasites and observations on the gametocytemia in schoolchildren living in a malaria-endemic area of Mozambique. *American Journal of Tropical Medicine and Hygiene,* 52: 50-59.

Mount, D.L., Nehlen, B.L., Patchen, L.C. & Churchill, F.C. (1989). Adaptations of the Saker-Solomons test: simple, reliable colorimetric field assays for chloroquine and its metabolites in urine. *Bulletin of the World Health Organization*, 67, 295-300.

Paul RE, Doerig C, Brey PT Erythropoiesis and molecular mechanisms for sexual determination in malaria parasites. UBMB Life 2000;49:245-8

Price, R.N., Nosten, F., Luxemburger, C., ter Kuile, F.O., Chongsuphajaisiddhi, T. & White, N.J. (1996). The effects of artemisinin derivatives on malaria transmissibility. *Lancet*, 347, 1654-1658.

Price, R.N., Nosten, F., Simpson, JA., Luxemburger, C., Phaipunm L., ter Kuile, F.O., Van Vugt, M.A., Chongsuphajaisiddhi, T. & White, N.J. (1996). Risk factors for gametocyte carriage in uncomplicated malaria. *American Journal of Tropical Medicine and Hygiene*, 60, 1019-1023.

Robert, V., Awono-Ambene, H.P., Le Hesran, J.Y. & Trape J. F. (2000). Gametocytemia and infectivity to mosquitoes of patients with uncomplicated *Plasmodium falciparum* malaria attacks treated with chloroquine or sulfadoxime plus pyrimethamine. *American Journal of Tropical Medicine and Hygiene*, 62, 210-216.

Targett, G., Drakeley, C., Jawara, M., von Seidlein, L., Coleman, R., Deen, J., Pinder, M., Doherty, T., Sutherland, C., Walraven, G., and Milligan, P. Artesunate reduces, but does not prevent, post-treatment transmission of *Plasmodium falciparum* to *Anopheles gambiae*. In press.

Thompson, D. (1911). Research into the production, life and death of crescents in malignant tertian malaria in treated and untreated cases by a enumerative method. *Ann. Trop Med. Parasitol.* 5,57-81*.*

Thompson, D. (1914) The origin and development of gametes (crescents) in malignant tertian malaria: some observations on flagellation. *Ann. Trop Med. Parasitol.* 8: 85-104.

Shute, P.G. and Maryon M. (1951). A study of gametocytes in a West African strain of *Plasmodium falciparum*. *Trans. Roy. Soc. Trop. Med. Hyg.* 44; 421-438.

Schneweis, S., Maier, W.A. Seitz, H.M. (1991) Haemolysis of infected erthrocytes- a trigger for formation of *Plasmodium falciparum* gametocytes? Parasitological research 77 ;458-460.

von Seidlein, L., Bojang, K., Jones, P., Jaffar, S., Pinder, M., Obaro, S., Doherty, T., Haywood, M., Snounou, G., Gempeli, B., Gathmann, I., Royce, C., McAdam, K. & Greenwood, B. (1998). A randomised controlled trial of artemether/benflumetol, a new antimalarial and pyrimethamine/sulfadoxine in the treatment of uncomplicated falciparum malaria in African children. *American Journal of Tropical Medicine and Hygiene*, 58, 638-644.

von Seidlein, L., Milligan, P., Pinder, M., Bojang, K., Anyalebechi, C., Gosling, R., Coleman, R., Ude, J.I., Sadiq, A., Duraisingh, M., Warhurst, D., Alloueche, A., Targett, G., McAdam, K., Greenwood, B., Walraven, W., Olliaro, P. & Doherty, T. (2000). Efficacy of artesunate plus pyrimethamine-sulfadoxine for uncomplicated malaria in Gambian children: a double-blind, randomised, controlled trial. *Lancet*, 355, 352-357.

von Seidlein, L., Clark S., Alexander N., Walraven, G and Pinder M. Treatment uptake of antimalarials by Plasmodium infected individuals in rural Gambia, West Africa submitted Dec 2000.

WHO (1973). Chemotherapy of malaria and resistance to antimalarials. Geneva: World Health Organization, Technical Report Series no. 529.

WHO (1993). Implementation of the Global Malaria Control Strategy. Geneva: World Health Organization, Technical Report Series no. 839.

**B3 Details of study design and investigations**

This section is designed to give the Committees sufficient information to see clearly and quickly the scientific and ethical aspects of the study design. Some parts will not be relevant to all studies. For studies at MRC Laboratories, please discuss data management arrangements with the Head of Computing. For clinical studies, please discuss the clinical service commitments with the Clinical Director. If you have questionnaires or consent forms prepared, please attach these to the application.

1. **What type of study design is proposed (eg case control, prospective cohort, randomised controlled trial, descriptive etc)**

Randomised controlled trial

1. **What is the proposed size of the study (this may relate to patients, cases, controls, survey subjects, laboratory samples etc, as appropriate).**

We will screen up to 1500 individuals aged ≥ 6 months to recruit 300 people with asymptomatic *P. falciparum* malaria infection with >20 parasites/μL blood willing to participate in the study

1. **Please describe the statistical considerations and sample size calculations involved in determining the size of the study.** (If you do not have access to statistical advice, please consult the MRC Laboratories Statistics Department.)

A survey conducted in the study area in June 2000 has shown a prevalence of *P. falciparum* asexual parasitaemia and gametocytaemia of 30% and 8%, respectively (PINDER and JAWARA, ongoing study). This rate might increase to 15% if molecular assay (RT-PCR) is used for the detection of gametocytes. Assuming a gametocyte rate of at least 15% in the control group, a sample size of 71 participants in each group will have 80% power at 95% significance level to detect a threefold or more increase in gametocyte rate in the Fansidar® alone group seven days after treatment and gametocyte rate of 1% or less in the Fansidar® plus single dose artesunate group 28 days after treatment compared to that in the control group. In order to allow for drop out a total of 100 participants per group will be recruited. Assuming a prevalence of *P. falciparum* asexual parasitaemia of 30% in the study area at this time of the year and allowing for refusal of participation by eligible participants about 1500 individuals will be screened to participate in the study.

**For studies involving human subjects:**

1. **How and where will the study subjects (cases, controls, etc) be selected? Has it been confirmed that they are not already involved in other studies?**

The study subjects will be selected from the demographic surveillance database of the Farafenni Field Station. This will be co-ordinated by the Head of the Station to ensure that the subjects are not involved in other studies.

**What inclusion/exclusion criteria will be applied?**

**Inclusion criterion**

- Individuals with *P. falciparum* malaria infection but without symptoms suggestive of malaria or pyrexia (axillary temperature ≥ 37.5°C) after two screening sessions conducted two days apart.

**Exclusion criteria**

- Pregnant women.
- Children weighing less than 5 kg.
- Individuals with acute medical conditions.
- History of chloroquine use, hypersensitivity to any of the study drugs or their use within the past 4 weeks.

1. **How will informed consent be obtained?**

Meetings will be held in the villages to explain in the local language the objectives and methods of the study, the inconveniences and advantages involved and to answer questions from the residents. Consent of the villages will be obtained before the commencement of the study. Residents aged 6 months and above will be screened for participation in the study after they or their accompanying parents/relatives have signed a written informed consent form **(Appendix 1)**.

1. **What samples, if any, will be taken and what investigations will be conducted?**

Only finger prick blood samples will be taken. The investigations will be PCV determination, thick blood films, filter paper (FP) collection and Tri-reagent for the detection of malaria parasites by microscopy and molecular assays. The samples/investigations to be carried out at the various surveys are summarised in the table below:

**Summary of surveys and investigations to be carried out**

|  | Activity | Investigation | | | |
| --- | --- | --- | --- | --- | --- |
| Day |  | Blood smear | Tri | FP | PCV |
| D-2 | Screening and treatment of acute cases |  |  |  |  |
| D-1 | Slide reading |  |  |  |  |
| D0 | Feedback to villagers randomisation, treatment of participants |  |  |  |  |
| D3 | Follow up survey |  |  |  |  |
| D7 | Follow up survey |  |  |  |  |
| D14 | Follow up survey |  |  |  |  |
| D28 | Follow up survey |  |  |  |  |
| D56 | Follow up survey & treatment of control group |  |  |  |  |
| **Total** |  | **6** | **6** | **6** | **2** |

1. **Will treatment be given? YES**

**If yes:**

**Nature of treatment(s)**

Fansidar® is composed of 25 mg pyrimethamine and 500 mg sulfadoxine. Artesunate is an artemisinin derivative. Lactose tablets (which is inert) will be used as the comparator drug (for the control group).

**For drugs: dosage and duration of treatment**

Adults and those weighing ≥ 50 kg will receive 3 tablets of Fansidar® while children will receive half a tablet if their body weight is less than 10 kg and an additional quarter tablet for every 5-kg increase in weight. For artesunate adults and those weighing ≥ 50 kg will receive 4 tablets (50-mg tablet size) while the dose for children will be 4 mg/kg body weight. Fansidar® and its combination with artesunate will be administered as single dose. A single dose of lactose tablets will be given to participants in the control group.

**Person(s) responsible for administering treatment**

Clinical Epidemiologist (PI)

1. **For questionnaires/interviews, who will be conducting these?**

Clinical Epidemiologist and field assistants

1. **Who will be primarily responsible for the statistical design and analysis?**

PI/Paul Milligan

1. **Who will be primarily responsible for data management?**

PI/Data Manager at Farafenni Field Station

**C Ethical issues**

**Please highlight any potential ethical issues and how you propose to deal with these. What outcomes and benefits will derive from the study? How will the results of the study contribute to the health of the people of the Gambia?**

This section, together with B3 is particularly important to the Ethical Committee and should be comprehensive. Please continue on a separate sheet if necessary.

Fansidar® sometimes causes severe cutaneous reactions and blood dyscrasias such as agranulocytosis and haemolytic anaemia when used for long-term prophylaxis. These side effects, which are due to the sulphonamide component of the mixture, are rare with therapeutic doses. Clinics will be held in the villages for the period of the study and any participant with adverse reactions or other medical problems will be given the appropriate treatment or referred for further management free of charge. Participants in the Fansidar® and Fansidar® plus single dose artesunate groups who present with symptoms and parasitological confirmation of malaria during the follow-up period will receive rescue medication with quinine while those in the control group will be rescued with chloroquine and Fansidar®. At the end of the study individuals with parasitaemia in the control group will be treated with chloroquine and Fansidar®.

The study will provide data for a clearer understanding of the response of the human reservoir of gametocytes to drugs that have potential for use as transmission reducing agents. Such information will be vital in policy decisions concerning transmission reduction through mass drug administration.

**D Resources Required**

**D1 Summary and cost**

The Committees need to be reassured that the resource implications of the study have been fully considered and that the resources are available or are being sought to complete the study. Please refer to the following guidance in completing the table overpage.

For all cost categories please indicate the source of funding. For MRC projects this may be existing MRC budget(s) if they contain sufficient uncommitted funds (if so please give budget code and title), or an external source. If the project has not already been fully costed for a funding application, please discuss the resources needed with the Personnel and Finance Manager or the Administrative Director. As a brief guide:

**Staff**  - please include existing support staff for information, and provide costs for any new staff required to complete the study. For new staff to be employed by MRC, full staff costs (including social security contributions, recruitment etc) should be obtained from the Personnel and Finance Manager.

**Consumables** – these include all laboratory consumables, medicines and other clinical supplies, questionnaire production, computer consumables, specialist stationery and other supplies particular to the project. Freight costs should be included. The Laboratory Manager will advise on costs.

**Capital and minor equipment** – this should include the cost of any equipment that needs to be bought, replaced or repaired for the project. Freight and installation costs should be included. For projects at the MRC Laboratories, please discuss the availability of laboratory equipment with the Laboratory Manager.

**Transport** – if MRC transport is required, you will need to discuss with the Transport Manager the frequency and length of journeys, what is the most cost-effective way of meeting the need, and whether the resources will be available (vehicles, drivers etc). You should also allow for any local public transport costs for staff or study subjects, and night allowances for staff.

**Other** – this may include, particularly for externally-funded projects, accommodation costs (residential and working space), clinical fees and other overhead charges, communication costs, training, meeting costs, library. Please discuss the availability of laboratory accommodation with the Laboratory Manager and of residential accommodation with the Personnel and Finance Manager (short stays) or Administrative Director (longer term).

| **Type** | **Details** | **Cost £** | **Source(s) of funding – external and internal** |
| --- | --- | --- | --- |
| Support staff already in post | 1 Clinical Epidemiologist  2 Laboratory Assistant  2 Field Assistants  1 Driver  1 Data Entry Clerk |  | Core staff |
| New staff required | 4 Village Assistants  (To be paid total of £100 for 5 months) | 500 |  |
| Consumables | Laboratory Supplies and Equipment  Stationery and Computing  **(Appendix 4)** |  |  |
| Existing Capital or Minor equipment  to which access is needed | Microhaematocrit centrifuge  Compound Microscopes  PCR machine  Generator (for field use) |  |  |
| New Capital Equipment required (ie >£3,000) | None |  |  |
| New Minor Equipment required | None |  |  |
| Transport and local travel | 2 motor bikes 125 (available)  1 Landrover for fieldwork for 4 months and Travel Farafenni-Fajara 4 times |  |  |
| Other (specify) |  |  |  |

**D2 Sources of funds**

If external funds have been or will be sought, please state the progress of the application(s).

MRC malaria core programme.

**Appendix 1**

**MRC FARAFENI FIELD STATION**

**Information sheet and Consent form for asymptomatic malaria treatment study**

The following should be explained to the participant (or the parent/guardian of the participant in the case of a child) in the language s/he understands. The attached consent form should be signed by only those who have agreed to participate in the study.

Malaria is a common cause of disease and death in The Gambia. It is caused by the bite of mosquitoes carrying malaria parasites, which they had earlier picked from infected people during a blood meal. Some people have mild or severe malaria illness while others just carry the parasites in their blood without being ill. The MRC is carrying out this study to find the appropriate treatment to reduce the parasites that infect mosquitoes and are responsible for spreading the disease. If we know this, then we will be able to offer treatment that can reduce the spread of malaria.

If you are participating in this study, we will ask you to do the following:

We will invite you to our clinic in the village and interview you about your/your child's state of health, examine you/your child and take a small quantity of finger prick blood from you/ your child (less than one-third teaspoonful of blood) to look for malaria parasites. We will also collect a urine sample from you/your child to check whether you have used chloroquine recently. If we find malaria parasites, and the urine test shows no trace of chloroquine then we will ask you/your child to join the study. You or your child may receive Fansidar® alone, the combination of Fansidar® and single dose artesunate or placebo. Fansidar® is a widely used treatment for malaria in the Gambia. We have used artesunate to treat many children with malaria and found it to be safe and very effective. None of the children who took these drugs had any bad experiences. We do know that a very small number of people who take these drugs (perhaps one person out of 10,000) may get a rash or problems with their blood. Clinics will be held in the villages for the period of the study and any participant with medical problems will be given the appropriate treatment free of charge.

A field worker will visit you/your child at home on 3, 7, 14, 28 and 56 days after treatment to check you are well and to repeat the finger prick blood sampling on you/your child. This is the only way we can know if the treatment is able to suppress the stage of the malaria parasites that the mosquitoes pick from humans and later transmit to others.

All the information you give to us will be treated as confidential. You do/your child does not have to join this study. If you do or your child does join, you/your child can withdraw whenever you wish. Withdrawal will not affect you or your family's health care. If you have any questions or queries at any time during the study, the study team will be happy to talk to you about them.

**CONSENT FORM**

The information sheet has been read to me/I have read and understood the information sheet and I have had the chance to ask questions about the study.

I understand that I will be interviewed about my/my child's state of health, examined and a small quantity of finger prick blood taken to look for malaria parasites. Urine sample will also be collected to check for previous chloroquine use. If malaria parasites are found and the urine test shows no trace of chloroquine then I/my child will be asked to join the study.

I understand that I/my child may receive Fansidar® alone, the combination of Fansidar® and single dose artesunate or placebo. A field worker will visit me/my child home 3, 7, 14, 28 and 56 days after treatment to check that we are well and to repeat the finger prick blood sampling on me/my child.

I understand that if I/my child develop(s) any medical problems during the study, treatment will be provided free of charge. Information about me/my child will remain confidential and will be used only for the purposes of the study.

I understand that I do/my child does not have to take part in the study, and that we can leave the study at any time, and this would not affect the health care I or my immediate family may receive while the trial is going on.

Name of participant_________________________Signature/Right thumbprint __________

*(For children ≤ 16 years old)*

Name of parent/guardian_________________________________

Signature/Right thumbprint __________________________

Date (dd/mm/yy) ___/___/___ Village Code |__|__|__| Study Number |__|__|__|__|

I confirm that I have explained the study to the participant/parent/guardian in the language they understand and I believe he/she understood and is participating/his or her child is participating out of his/her free will.

Name of Field Assistant (block letters)…...……………...…............…………….

Signature of Field Assistant: .................................................. Date ___/___/___
